# The Widened Pipe Model of plant hydraulic evolution

**DOI:** 10.1073/pnas.2100314118

**Published:** 2021-05-26

**Authors:** Loren Koçillari, Mark E. Olson, Samir Suweis, Rodrigo P. Rocha, Alberto Lovison, Franco Cardin, Todd E. Dawson, Alberto Echeverría, Alex Fajardo, Silvia Lechthaler, Cecilia Martínez-Pérez, Carmen Regina Marcati, Kuo-Fang Chung, Julieta A. Rosell, Alí Segovia-Rivas, Cameron B. Williams, Emilio Petrone-Mendoza, Andrea Rinaldo, Tommaso Anfodillo, Jayanth R. Banavar, Amos Maritan

**Affiliations:** ^a^Dipartimento di Fisica e Astronomia G. Galilei, Istituto Nazionale di Fisica Nucleare, Università di Padova, 35131 Padova, Italy;; ^b^Laboratory of Neural Computation, Istituto Italiano di Tecnologia, 38068 Rovereto, Italy;; ^c^Instituto de Biología, Universidad Nacional Autónoma de México, Ciudad de México 04510, Mexico;; ^d^Departamento de Física, Universidade Federal de Santa Catarina, Florianópolis-SC 88040-900, Brazil;; ^e^Dipartimento di Matematica Tullio Levi-Civita, Università di Padova, 35121 Padova, Italy;; ^f^Department of Integrative Biology, University of California, Berkeley, CA 94720-3140;; ^g^Department of Environmental Science, Policy and Management, University of California, Berkeley, CA 94720-3140;; ^h^Instituto de Investigación Interdisciplinario (I^3^), Universidad de Talca, Campus Lircay, Talca 3460000, Chile;; ^i^Dipartimento Territorio e Sistemi Agro-Forestali, Università di Padova, Legnaro 35020, Italy;; ^j^Departamento de Ecología Evolutiva, Instituto de Ecología, Universidad Nacional Autónoma de México, 04510 Ciudad de México, Mexico;; ^k^Faculdade de Ciências Agronômicas, Universidade Estadual Paulista, São Paulo 18603970, Brazil;; ^l^Biodiversity Research Center, Academia Sinica, Taipei 11529, Taiwan;; ^m^Laboratorio Nacional de Ciencias de la Sostenibilidad, Instituto de Ecología, Universidad Nacional Autónoma de México, 04510 Ciudad de México, Mexico;; ^n^Channel Islands National Park, Ventura, CA 93001-4354;; ^o^Santa Barbara Botanic Garden, Santa Barbara, CA 93105-2126;; ^p^Department of Biological Sciences, Northern Arizona University, Flagstaff, AZ 86011-7014;; ^q^Laboratory of Ecohydrology, IIE/ENAC, Ecole Polytechinque Fédérale de Lausanne, 1015 Lausanne, Switzerland;; ^r^Dipartimento Ingegneria Civile Edile e Ambientale, Università di Padova, 35131 Padova, Italy;; ^s^Department of Physics, University of Oregon, Eugene, OR 97403;; ^t^Institute for Fundamental Science, University of Oregon, Eugene, OR 97403

**Keywords:** plant hydraulics, xylem, Pareto optimality, allometry, adaptation

## Abstract

Shaping global water and carbon cycles, plants lift water from roots to leaves through xylem conduits. The importance of xylem water conduction makes it crucial to understand how natural selection deploys conduit diameters within and across plants. Wider conduits transport more water but are likely more vulnerable to conduction-blocking gas embolisms and cost more for a plant to build, a tension necessarily shaping xylem conduit diameters along plant stems. We build on this expectation to present the Widened Pipe Model (WPM) of plant hydraulic evolution, testing it against a global dataset. The WPM predicts that xylem conduits should be narrowest at the stem tips, widening quickly before plateauing toward the stem base. This universal profile emerges from Pareto modeling of a trade-off between just two competing vectors of natural selection: one favoring rapid widening of conduits tip to base, minimizing hydraulic resistance, and another favoring slow widening of conduits, minimizing carbon cost and embolism risk. Our data spanning terrestrial plant orders, life forms, habitats, and sizes conform closely to WPM predictions. The WPM highlights carbon economy as a powerful vector of natural selection shaping plant function. It further implies that factors that cause resistance in plant conductive systems, such as conduit pit membrane resistance, should scale in exact harmony with tip-to-base conduit widening. Furthermore, the WPM implies that alterations in the environments of individual plants should lead to changes in plant height, for example, shedding terminal branches and resprouting at lower height under drier climates, thus achieving narrower and potentially more embolism-resistant conduits.

## Significance

For most of its path through plant bodies, water moves in conduits in the wood. Plant water conduction is crucial for Earth’s biogeochemical cycles, making it important to understand how natural selection shapes conduit diameters along the entire lengths of plant stems. Can mathematical modeling and global sampling explain how wood conduits ought to widen from the tip of a plant to its trunk base? This question is evolutionarily important because xylem conduits should widen in a way that keeps water supply constant to the leaves as a plant grows taller. Moreover, selection should act on economy of construction costs of the conducting system. This issue is ecologically important because it helps suggest why climate change alters vegetation height worldwide.

Water transport through plants is a key driver of the carbon and other biogeochemical cycles (1–3) and is a crucial link in plant adaptation to climate and vegetation response to climate change (4–9). The water conducting cells of plants, xylem conduits, widen with distance from the stem tip, and, therefore, taller plants have wider conduits (6, 10–12). Xylem conduits are of two main types: tracheids, found in most gymnosperms, and vessels, found in most flowering plants. Tracheids have intact cell membranes, so water must flow from cell to cell through these membranes. Vessels are made up of cells aligned vertically end to end, with the cell membranes dissolved between successive members, forming a tube. Whatever their differences in structure, wider conduits are beneficial because they conduct more water. Tip-to-base widening is expected to help maintain conductance per unit leaf area constant as an individual plant grows taller, counterbalancing the resistance that would otherwise accrue with increasing conductive path length the individual grows (2, 13). Wider conduits, however, are more vulnerable to embolisms caused by cold and likely drought (8, 14–18) and cost more in terms of carbon for a plant (ref. 1; cf. ref. 19). Embolisms in the xylem even affect transport of photosynthates in the phloem (8, 20). This means that as trees grow taller, conductance, embolism vulnerability, and carbon costs must interrelate in a delicate evolutionary balance.

Because of the importance of this balance in plant hydraulic evolution and in forest reactions to climate change (3, 6, 21–23), an important goal of plant biology is to construct models that predict how and why plants deploy conduit diameters throughout their bodies (1, 2, 17, 24–26). Some models predict that conduits should be of uniform diameter (27, 28), while others predict that they should widen tip to base (1, 2, 13, 24, 29, 30). But even current models include untested assumptions and large numbers of parameters, making it difficult to identify the biological causes of the predictions they make. For example, some invoke Da Vinci’s rule, the largely untested assumption that the summed wood area of the twigs is the same as that at the base (24, 26). Other models depict plant conduits as branching as they do in mammalian circulatory systems, but whether this happens along the entire stem in plants is unclear (30–33). There is an expectation that conduit diameter *D* should widen with distance from the stem tip *L* following a power-law (*D ∝ L*^*b*^), but there is no agreement on the value of *b,* the conduit widening exponent (1, 2). Furthermore, even though within-individual tip-to-base conduit widening has been confirmed in a handful of species (34–36), and the scaling of conduit diameter with plant size across species is consistent with it (6, 10–12, 34), the expectation that conduits should widen similarly within stems across terrestrial vascular plant lineages and habits has yet to be empirically confirmed. Here we present the Widened Pipe Model (WPM), which correctly predicts the form of tip-to-base conduit widening across the span of plant size, life form, and habitat across the terrestrial plant phylogeny.

## Results

### The WPM.

Our general theory predicts the form of tip-to-base conduit widening invoking a trade-off between two opposing and essential evolutionary drivers: selection minimizing fluid dynamic resistance *R* (2), while at the same time minimizing the rate of tip-to-base conduit widening *W*. We modeled conduits as independent tubes that are continuous tip to base to predict a profile of tip-to-base conduit widening that should be universal along plant stems.

### Resistance Cost *R*.

If resistance increased as stems grew longer, plants would be at a constant disadvantage as they grow (13). As a result, selection should favor a widening profile that minimizes hydraulic resistance. The laminar flow of a Newtonian fluid through a cylindrical pipe can be described by the Hagen−Poiseuille law (37–39), which represents an exact solution of the general Navier−Stokes equations. The volume flow rate *Q* of a liquid through a pipe is given by

$$Q=\frac{\left|\Delta P\right|\pi r^{4}}{8\mu L},$$ [1]

where μ is the fluid viscosity, ΔP is the pressure gradient between the tip and the base of the pipe, *L* is the total length, and *r* is the internal radius. By analogy with Ohm’s law for electrical circuits, we can define the resistance, R, for the pipe as

$$R=\frac{\left|\Delta P\right|}{Q}=\frac{8\mu L}{\pi r^{4}}.$$ [2]

For pipes that vary in their diameter along their lengths, the Hagen−Poiseuille law is valid only for sections of infinitesimal lengths, for each of which the radius, *r*(*h*), is approximately constant. For a circular cross-sectional area, $\sigma \left(h\right)=\pi \;r{\left(h\right)}^{2}$, we can write the infinitesimal resistance as

$$dR\left(h\right)=\frac{K\mu }{\sigma^{2}\left(h\right)}\;dh,$$ [3]

where K=8π (40). Quite generally, the total resistance of a tree’s hydraulic pathway is the integral of dR(h) along the whole xylem path length,

$$R=\mu K\overset{h_{M}}{\underset{h_{0}}{\int }}\frac{1}{\sigma^{2}\left(h^{'}\right)}dh^{'},$$ [4]

where $h_{0}$ and $h_{M}$ are the minimum and the maximum distances from the tip, respectively; $h_{M}$ is a measure of tree height, whereas $h_{0}$ is the length of the conductive units (vessel elements, tracheids, or hydroids). We will define a rescaled resistance Ω=R/μK for use in some of our calculations below. Selection should thus minimize hydraulic resistance, but only insofar as embolism risk and carbon cost are also minimized, considerations that are reflected by the widening cost.

### Widening Cost *W*.

The widening cost penalizes widening that is too rapid moving from the stem tip to the base, reflecting two parallel selection pressures. The first is minimization of embolism vulnerability. Wider conduits are potentially more vulnerable to embolism (14–17, 41–43). At the same time, xylem water potential is most highly negative near the stem tip, becoming less so toward the base (44). High tensions promote embolism, so narrow diameters at the stem tip would mean that conduits are more resistant where the risk of embolism is high (24, 25). Selection should thus minimize the rate of conduit widening tip to base. The second pressure is carbon cost. A wider conduit costs more to construct than a narrow one (ref. 1; cf. refs. 19, 20, 30, and 45), and wider conduits require more photosynthates for embolism repair and osmotic regulation of conduction. Selection should therefore favor tip-to-base profiles that minimize carbon cost for a given unit of conductance. A profile that widens too fast, reaching practically its final cross-section very close to the tip, would be approximately cylindrical for most of its length. Such a cylindrical profile would lead to minimal hydraulic resistance but would also represent the maximal carbon cost and the highest embolism risk. Natural selection should simultaneously minimize *W* and *R*, balancing these two competing vectors of selection. The cost associated with the widening rate is captured with a functional of the form

$$W=\overset{h_{M}}{\underset{h_{0}}{\int }}f\left(\dot{\sigma }\left(h\right)\right)dh,$$ [5]

where $\dot{\sigma }\left(h\right)=d\sigma \left(h\right)/dh$, and the function *f* can be expanded as

$$f=a_{1}\dot{\sigma }\left(h\right)+a_{2}{\dot{\sigma }}^{2}\left(h\right)+\ldots a_{n}\;{\dot{\sigma }}^{n}\left(h\right).$$ [6]

The first term in Eq. **6** gives the trivial contribution to *W,* $a_{1}\left(\sigma \left(h_{M}\right)-\sigma \left(h_{0}\right)\right)$, which contains only the total variation of the xylem cross-section with no information regarding how this contribution changes as a function of the tree height *h*. To make the analytical treatment feasible, we considered only the first nontrivial term $a_{2}{\dot{\sigma }}^{2}\left(h\right)$,

$$W=a_{2}\overset{h_{M}}{\underset{h_{0}}{\int }}{\dot{\sigma }}^{2}\left(h\right)dh\text{.}$$ [7]

Conduit carbon cost is taken to be proportional to the total surface area of a xylem conduit and is a measure of the energy needed to build the conduit walls. We use the following formula for carbon cost:

$$C\;=\overset{h_{M}}{\underset{h_{0}}{\int }}dh\;2\pi \sqrt{\frac{\sigma \left(h\right)}{\pi }}.$$ [8]

### How Selection Should Act in the Context of the *R−W* Trade-off: Analytical Solution.

The optimal *R−W* trade-off can be solved in the context of multiobjective Pareto optimization (46–49), which, in turn, can be converted into single-objective functions (50). The optimal solution of the *R−W* trade-off can be found by minimizing a single objective, which is a linear combination of the fluid conductance *R* and the widening cost *W.* The parameter of the linear combination is the Lagrange multiplier, λ. We seek to minimize, with respect to all possible profiles σ(h), of R+λW, or, equivalently, of $F\left[\sigma ,\;\dot{\sigma }\right]=\left(R+\lambda W\right)/\mu K$. $F\left[\sigma ,\;\dot{\sigma }\right]$, as follows:

$$F\left[\sigma ,\;\dot{\sigma }\right]=\overset{h_{M}}{\underset{h_{0}}{\int }}L\left(\sigma \left(h\right),\dot{\sigma }\left(h\right)\right)dh,$$ [9]

where the Lagrangian $L\left(\sigma \left(h\right),\dot{\sigma }\left(h\right)\right)$ has the following form:

$$L\left(\sigma \left(h\right),\dot{\sigma }\left(h\right)\right)=\;\frac{1}{\sigma^{2}\left(h\right)}+\;\frac{\alpha }{2}\;\dot{\sigma^{2}}\left(h\right),$$ [10]

and $\alpha =2a_{2}\lambda /\mu K>0$ is the first free parameter of our theory. Since $a_{2}$ enters only in the combination $a_{2}\lambda$, we can set $a_{2}$ to 1 without loss of generality.

The set of optima is made up of the solutions of multiobjective optimization problems (46–48). In our case, we have two objective functions, the hydraulic resistance *R* and the widening rate *W*, and thus the optimal front would correspond to a one-dimensional curve in the *R−W* space spanned by the free parameter α (or equivalently λ). This is discussed in *The Pareto Front*.

### Central Result of the WPM.

The main result of our theory is a closed-form analytical solution for the widening of a single conduit as a function of distance from the stem tip, *h*, as

$$\sigma \left(h\right)=\sigma_{M}\sqrt{\frac{h}{h_{M}}\left(2-\frac{h}{h_{M}}\right)}\equiv \sigma_{M}F\left(\frac{h}{h_{M}}\right),$$ [11]

where σ(h) is the cross-sectional area and $\sigma_{M}$ is the value of σ(h), when $h=h_{M}$, the distance from the stem tip to the base (see *Materials and Methods*). $F\left(x\right)=\;{\left[x\;\left(2-x\right)\right]}^{1/2}$ is a scaling function. Crucially, Eq. **11** depends only on the scaled variables $\sigma \left(h\right)/\sigma_{M}$ and *h/h*_*M*_, implying that the conduit widening profile should be universal across terrestrial plants, when conduit cross-sectional area and distance from the stem tip are measured in units of $\sigma_{M}$ and $h_{M}$, respectively. Eq. **11** exhibits power-law behavior ($D\propto h^{0.25}$) only close to the stem tip. Farther down the trunk, conduit cross-sectional area gradually departs from pure power-law behavior, being narrower than expected and reaching a maximum deviation of around 30% from power-law expectations close to the tree base (1, 36).

### Data Collection.

**Fig. 1.**
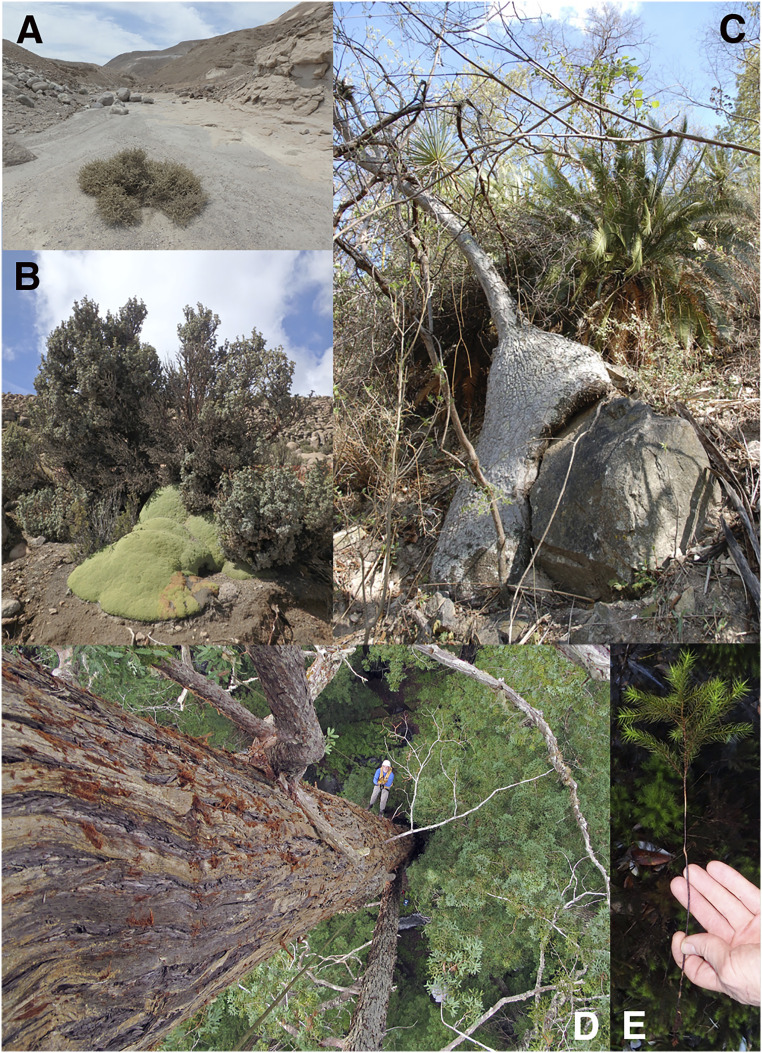
Examples of habit, habitat, and phylogenetic diversity of the sampled species. (*A*) *A. imbricata* growing on the fringe of the hyper-arid core of the Atacama Desert, Chile, where it is virtually the only plant growing. (*B*) *P. tarapacana* at 5,000 m above sea level, well above the elevational limit for virtually all other trees, in the Chilean Andes. (*C*) The arborescent monocot *B. olsonii* and the cycad *D. planifolium* growing in tropical dry forest in southwestern Puebla State, Mexico. (*D*) Our sampling included individuals of the world’s tallest trees, including *S. sempervirens* growing in California, with coauthor T.E.D. providing scale. Photo credit: Anthony R. Ambrose, University of Califorina, Berkeley. (*E*) We included a nonvascular plant, the giant moss *D. dendroides* from the temperate rainforest of Patagonian Chile.

To test our predictions, we collected tip-to-base conduit diameter data from 103 individuals across terrestrial vascular plant orders and life forms, from the world’s tallest trees to shrubs, cacti, and vines, from temperate and tropical rainforests to the world’s driest desert and freezing alpine habitats. Our sampling included the tallest species of trees in the world, including the California coast redwood *Sequoia sempervirens*, three individuals of which were over 100 m tall. Likewise, we sampled individuals over 90 m tall of the giant sequoia *Sequoiadendron giganteum*, and of mountain ash *Eucalyptus regnans*, the tallest flowering plant. These tall trees were all from cool, moist temperate rainforests; the smallest shrubs we sampled were an *Atriplex imbricata* 1.4 m tall, growing on the fringe of the hyperarid core of the Atacama Desert, at one of the driest localities on Earth that supports plant life, and a *Myrothamnus flabellifolia* 1.04 m tall, a resurrection plant from parched Namibian drylands. We sampled a nonvascular plant, a giant moss *Dendroligotrichum dendroides* 35 cm tall from the Patagonian rainforest, measuring its conductive cells, known as hydroids. In between these size and climate extremes, we sampled an array of shrubs and trees from tropical rainforests, tropical deciduous forests, tropical savannah, cloud forests, temperate rainforests, desert, Mediterranean woodland, and alpine vegetation from five continents. Fig. 1 shows some examples of this diversity.

### Benchmarking Data against Theory.

**Fig. 2.**
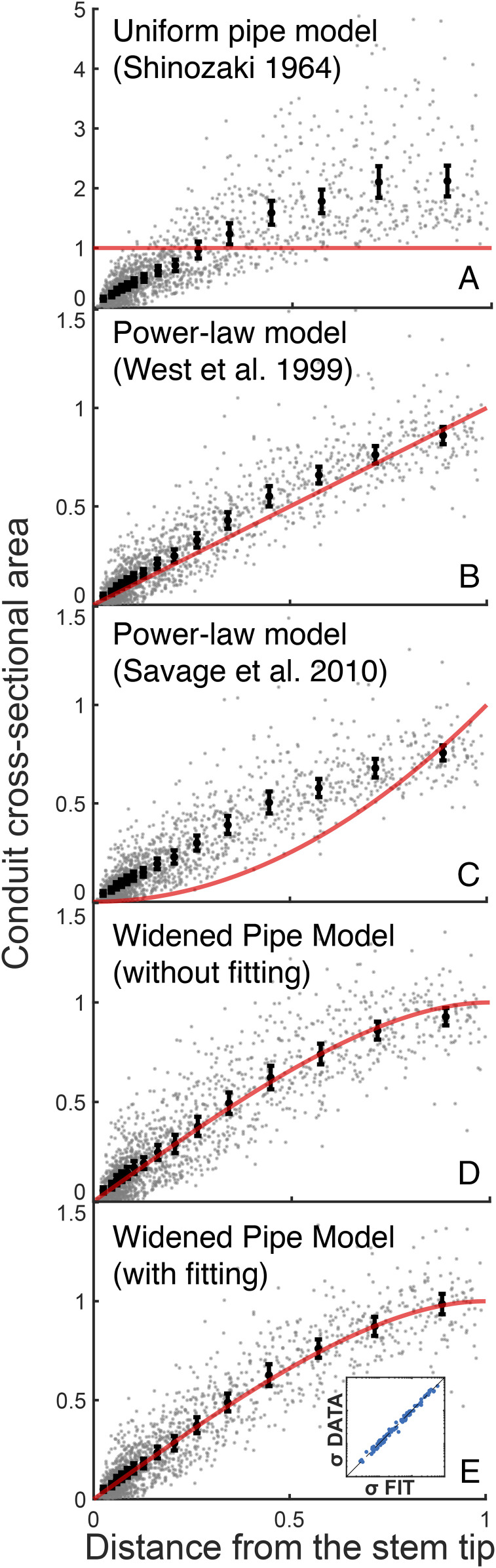
The WPM’s universal tip-to-base widening profile of xylem conduit diameter. Our theory predicts empirical conduit diameter profile data from across the terrestrial vascular plants as a function of distance from the stem tip. For each plant, we normalized both conduit cross-sectional area $[\sigma \;\left(h\right)/\sigma_{model}^{i}$] and distance from the stem tip, $\left[h/h_{M}^{i}\right]$, where $\sigma_{model}^{i}$ corresponds to the fitted model parameter for each plant, and $h_{M}^{i}$ is the height of the *i*th plant. Fitting parameters help take into account scatter about the *y* axis associated with different conduit types (tracheids versus vessels) and other factors (71). The bold points are averages over the 103 plants centered within 15 equally populated bins. The vertical bars denote 3 SDs from the mean. The red line depicts the analytical prediction of (*A*) the uniform pipe model (33) $\sigma \;\left(h\right)=\sigma_{Uniform\;pipe}^{i}$ ($\chi^{2}=136.64,\;p=1$), (*B*) the West et al. (2) model $\sigma \;\left(h\right)=\sigma_{WEST}^{i}{\left(h/h_{M}^{i}\right)}^{1/2}$ ($\chi^{2}=3.17,\;p<.001$), (*C*) the Savage et al. (24) model $\sigma \;\left(h\right)=\sigma_{SAVAGE}^{i}\left(h/h_{M}^{i}\right)$ ($\chi^{2}\approx 31.85,\;p=1$), and (*D*) our analytical prediction (Eq. **11**) in the case when $\sigma_{M}^{i}=\sigma_{Avg}^{i}$, with $\sigma_{Avg}^{i}$being the averaged cross-sectional area of the two lowest data points for each tree ($\chi^{2}=0.64,\;p<.001)$. (*E*) The analytical prediction when $\sigma_{M}^{i}$ is the fitting parameter to data. The averaged data points coincide strikingly with our analytical prediction (Eq. **11**) $\left(\chi^{2}=0.28,\;p<.001\right),$ highlighting the single universal curve of xylem conduit widening. *Inset* shows the cross-plot of the fitting parameters (the cross-sectional conduit areas at the base of the tree) along the horizontal axis and the measured cross-sectional areas along the vertical axis. The dashed line denotes the bisector. For clarity, we have stretched the horizontal axis with the transformation $X=x^{c}$, where c=0.5, to better highlight the power-law behavior near the stem tip and the deviation therefrom at higher values.

**Fig. 3.**
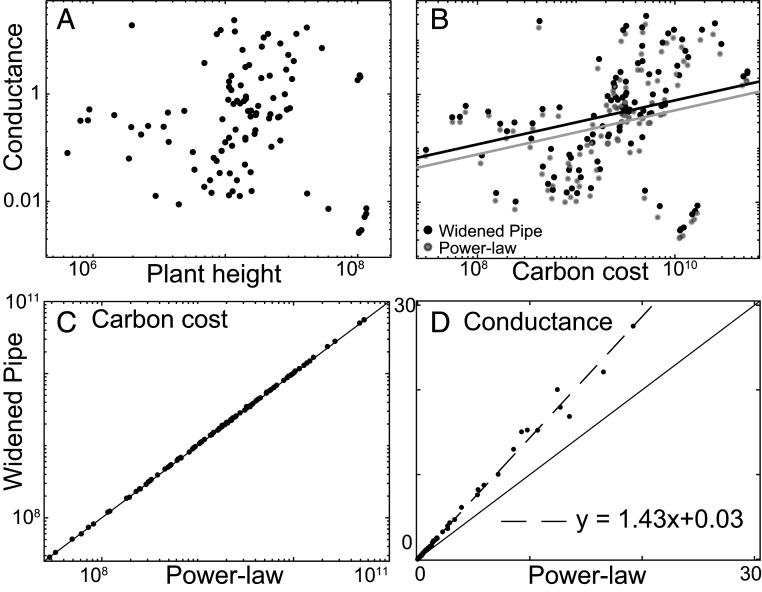
The WPM maximizes conductance given construction costs. (*A*) Conductance does not exhibit a systematic decrease with plant height, as shown in the log−log plot of μK⋅conductance (cubic micrometers), calculated for the universal conduit profile predicted by the WPM (Eq. **19**) versus plant height for the 103 plants ($R^{2}=10^{-3}$). Constant conductance with height growth means that leaves can maintain photosynthetic productivity despite increasing water transport distances. Here, μ is the fluid viscosity, and *K* is a geometrical factor (=8π for a circular cross-section). (*B*) Conductance versus carbon cost. We calculated conduit conductance and carbon cost for each of the 103 plants using two conduit profiles, the WPM, and a pure power-law (see Eqs. **16**–**19**) (2, 6, 72). The figure is a cross-plot of these quantities. The points for each of the 103 plants are paired, with WPM profile points conpsicuously above the power-law profile points, showing that conduits that widen following the WPM have distinctly higher conductance for the same carbon cost when compared to the widening following pure power-law behavior. Carbon cost is proportional to the external area of the conduits (square micrometers), while conductance is defined as the inverse of the rescaled Poiseuille resistance (cubic micrometers). These results implicate selection on carbon economy as an important factor shaping plant hydraulic systems. (*C* and *D*) Lower carbon costs per unit conductance as compared to pure power-law. (*C*) Total carbon cost of tip-to-base xylem conduit profiles for 103 plants. Each point corresponds to a given plant. That the points lie along the line with a slope of one (solid line) means that, for each plant, the carbon costs calculated for the two models are practically the same. (*D*) Total conductance of tip-to-base xylem conduit profiles for 103 plants. Each point corresponds to a given plant. The coordinate *x* represents the total conductance evaluated with the power-law profile, whereas the corresponding *y* coordinate is total conductance according to our prediction. These data were derived using a calculation without the assumptions A = $\sigma_{M}$ and vanishingly small $h_{0}.$ The fact the points lie on a dashed line of slope of about 1.43 indicates that our prediction conducts more efficiently than the pure power-law by about 43%. The solid line has a slope of one and is a guide to the eye.

Our empirical data from across the terrestrial vascular plants are in excellent accord with our predictions (Fig. 2), falling on the predicted universal curve *F* (*x*) = [*x* (2 − *x*)]^1/2^ ($\chi^{2}\left(15,N=15\right)=0.28,\;p<.001$; see Fig. 2*E*). Our prediction also results in conductance remaining approximately constant with height growth (Fig. 3*A*). In Fig. 3 *B*–*D*, we compare the carbon cost for the 103 plants studied using the prediction of our theory versus the results obtained using the pure power-law $\sigma \left(h\right)=A\sqrt{h/h_{M}}$, with *A* being a fitting parameter. We use the representative values of *h*_0_ = 4,000 μm for tracheids and *h*_0_ = 400 μm for vessel elements and hydroids. Here $h_{0}$ is the tracheid or vessel element length at the tip of the twig farthest from the base of the plant, and $h_{M}$ is tree height. The carbon costs of a conduit (assuming a tube of constant wall thickness running the length of a stem) for a given conductance is consistently lower when there is departure from pure power-law behavior. This result points to carbon economy as a powerful vector of natural selection shaping plant structure and function.

### The Pareto Front.

**Fig. 4.**
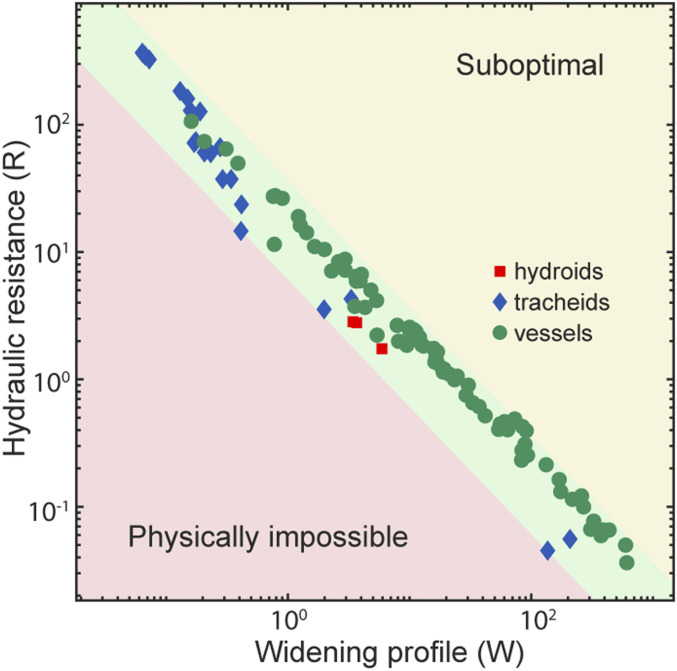
The WPM shows an optimal Pareto front between the conduit resistance cost *R* and the widening cost *W.* Points represent optimal solutions for each species. Physically accessible solutions fall in the yellow and green zones. The solutions in the green zone are optimal because there are no other solutions outperforming them simultaneously for both objective functions *R* and *W*. Optimal solutions exhibit an inverse relationship between the resistance and widening terms. Plants with tracheids, which are narrower conduits, tend to cluster together, having high resistance and a low tendency to widen, whereas plants with vessels, which are conduits that are typically wider than tracheids, have the highest values of widening and the lowest per-conduit resistances. The giant moss studied has hydroids, marked here in red.

Our theory predicts the Pareto front in the ln⁡Ω−ln⁡W objective space, that is,

lnΩ=−lnW+J, [12]

where *J* is a function that depends very weakly (logarithmically) on *h*_*M*_*/h*_*0*_; the observed empirical range of *h*_*M*_*/h*_*0*_ varies by three orders of magnitude, whereas *J* varies from ∼1.6 to 3.8 and can be considered almost constant. (See *Materials and Methods* for definitions of the relevant quantities.) The optimization process entails the simultaneous minimization of the conduit resistance and widening costs. The remarkable result of this optimization is that the universal profile, when suitably normalized, is independent of the relative weighting factor, λ, of the two costs. In consequence, the front of optimal trade-offs for the plants studied (Fig. 4) shows a conspicuous inverse relationship between the competing resistance and widening vectors of natural selection across species. Tracheids and hydroids, which are narrower conduits, have high resistance and a lower tendency to widen, whereas vessels have the highest values of widening and the lowest per-conduit resistances. Most strikingly, despite vast differences in structure, widening profiles are identical across these conduit types, and they all fall in the optimal green zone in Fig. 4.

## Discussion

That our model of a trade-off between just two vectors of natural selection should so accurately predict the conduit widening profile observed across terrestrial plants is striking and has far-reaching implications. Unlike our model, real conduits are neither perfect capillaries nor continuous tubes tip to base (17, 18). Moreover, water must pass through interconduit pit membranes (8, 17, 25, 26), which introduce resistance that we did not incorporate in our calculations. That our model predicts empirical patterns so well has the remarkable implication that additional sources of resistance must scale in concert with conduit resistance associated with tip-to-base conduit widening (51).

The WPM is very different from Shinozaki’s 60-y-old pipe model (27, 28), but we adapt the name to highlight some important similarities. The main, and crucial, difference is that the original pipe model assumed conduits of uniform tip-to-base diameter. Plant biologists quickly rejected Shinozaki’s pipe model because uniform diameters would lead to continual increases in resistance and drops in conductance with height growth. Our model, in contrast, predicts that conduits should widen with a finely regulated tip-to-base profile that buffers the increase in resistance with height growth in such a way that the conductance per unit leaf area, and thus photosynthetic productivity, can plausibly remain constant as a plant grows taller (Fig. 3*A*). Our results thus show how it is possible that a given leaf area can fix similar amounts of carbon despite height differences, and that trees can continue to produce similar amounts of wood per unit leaf area as they grow taller (52–56). A remarkable consequence is that forest productivity can therefore be estimated simply as the sum of leaf area, without taking plant height into account (57). Moreover, conduit widening should allow a sort of pipe model to hold in plant stems (33). For a given plant height, there is likely a constant number of parallel conduits per unit leaf area (58), as in the original pipe model. Among conspecifics of similar height but differing in basal trunk diameter, those with thicker trunks should have greater leaf area and thus more conduits, accounting for their thicker trunks, a prediction that, to our knowledge, has never been tested.

The finding that the observed tip-to-base widening profile across vascular plants achieves the same carbon cost with higher conductance as compared to a pure power-law profile (Fig. 3) points to carbon economy as an important vector of natural selection shaping plant conductive systems (3, 13, 45, 59). Given heritable variation between individuals in a population, individuals that invest less carbon for the same conductance will necessarily have more surplus carbon fueling further growth and reproduction compared to those that use more carbon. As a result, selection should potently economize carbon expenditure for a given performance (60), exactly in line with our findings. This result strongly calls into question the common notion that sapwood carbon costs increase per unit leaf area as plants grow taller (61). Our results suggest, instead, that it is more likely that selection favors a constant amount of metabolically active sapwood volume (not cross-sectional area) per unit leaf area with height growth: If heritable variants with greater carbon economy have greater fitness, as our results suggest, then it is unlikely that sapwood volume would proliferate massively per unit leaf area. Testing the prediction of leaf area−metabolically active sapwood volume isometry promises to be empirically laborious. However, if leaf area−sapwood volume scaling indeed proves isometric, it would imply a major realignment of theory regarding plant adaptation and even the causes of mortality in the face of climate change (59). As a result, testing the prediction of leaf area−metabolically active sapwood volume emerges as a priority for plant scientists.

The link between conduit diameter and height implies that changes in the environmental conditions experienced by individual plants should lead to changes in height (4, 6, 62). If narrower conduits are more embolism-resistant, then as climates dry in formerly moist areas, the maximum viable conduit diameter permitted by embolism risk should become narrower. Narrower conduits require shorter plants (35). Plants can become shorter by shedding terminal branches and resprouting at a lower height, which would achieve narrower, more embolism-resistant conduits (63). This prediction is consistent with the observation of the death of terminal branches in trees worldwide with climate change-induced drought (64, 65). Larger plants inevitably have wider conduits, which, in turn, are potentially more vulnerable to hydraulic failure (6, 7, 10–12, 14–16, 18). If wider conduits are more vulnerable, then all else being equal, large individuals should be preferentially vulnerable to mortality, consistent with the frequent death of large trees worldwide, as well as empirical evidence showing that larger plants, with their wider conduits, are more vulnerable to embolism (5, 6, 22, 23, 66, 67), potentially contributing to ongoing shifts in species distributions (68). Likewise, increasing plant height in the Arctic with global warming is consistent with the implication of our model that warmer temperatures should permit wider conduits and therefore taller plants (4, 6, 69). In this way, a web of opposing vectors of natural selection, maintaining constant conductance with minimal carbon cost, inescapably binds terrestrial plant size and hydraulics to one another (3), as climate change alters plant height and ecosystem services worldwide.

## Materials and Methods

### Plant Sampling: Size, Phylogenetic, Habit, and Climate Diversity.

Our theory predicts that all terrestrial vascular plants should be subject to the same pressures of natural selection postulated in our theory. Testing this prediction required sampling that adequately reflects terrestrial vascular plant diversity. In addition to the giant trees and desert shrub mentioned above (Fig. 1), our sampling included *Polylepis tarapacana*, a small tree growing at 5,000 m above sea level, well above the elevational limit for most trees. In addition to trees and shrubs, we included climbing lianas from just 60 cm to over 20 m long, as well as columnar cacti (*Marginatocereus marginatus, Pachycereus weberi*), a climbing palm (*Desmoncus orthoacanthos*), a fat-trunked “ponytail palm” (*Beaucarnea olsonii*), water-storing “bottle trees” (*Moringa drouhardii*), the giant tree poppy *Bocconia arborea*, the tree morning glory *Ipomoea wolcottiana,* arborescent monocots (*B. olsonii, Dracaena americana, Pandanus tectorius, Strelitzia nicolai*), bamboo (*Phyllostachys aurea*), and ancient cycads (*Dioon planifolium*). We sampled from virtually all vascular plant orders (*SI Appendix*, Fig. S1), including a spikemoss (*Selaginella*), club mosses (*Lycopodiella cernua*, *Lycopodium* sp.), a giant horsetail 5.3 m tall (*Equisetum myriochaeta*), a cycad, *Ginkgo biloba,* and the gnetophyte *Ephedra viridis*, as well as conifers including *Sequoia, Sequoiadendron, Pinus*, and *Podocarpus.* Within the angiosperms, we sampled *Amborella trichopoda*, the sister taxon to the rest of the flowering plants. In the remainder of the grade known as the basal angiosperms, we sampled from Austrobaileyales (*Illicium mexicanum*), Chloranthales (*Hedyosmum mexicanum*), Magnoliales (*Annona coriacea*), Laurales (*Siparuna thecaphora*), Canellales (*Drimys granadensis*), and Piperales (*Piper amalago*). We sampled from six families of monocots, including both lianescent and arborescent species. Among the noncore eudicots, we sampled Buxales (*Buxus sempervirens*), Trochodendrales (the vesselless *Trochodendron aralioides*), Proteales (*Roupala montana*), and Ranunculales (*B. arborea*). Within the core eudicots, we sampled from all orders that contain plants with appreciable accumulations of xylem. We additionally examined a nonvascular plant, the giant moss *D. dendroides*, which conducts water along its small “trunk” in cells called hydroids. In this way, our sampling spanned 67 plant orders, 86 families, 91 genera, 93 species, and 103 individuals (*SI Appendix*, Fig. S1).

### Field Sampling and Anatomical Methods.

We collected wood samples along the stem beginning at the shoot tip distal-most from the shoot base (13). Because conduit diameter changes quickly at the stem apex and then more slowly along the bole, we sampled densely near the shoot apex. For most species, we took samples every centimeter for the first 10 cm from the stem apex, then at 20, 30, 60, 120, 240, 480, and 960 cm, and so on, and at the base of each individual from the outer basal-most xylem, above buttresses when present (13). From each wood section, we cut thin wood cross-sections 10 μm to 30 µm thick with a sliding or rotary microtome, paraffin-embedding when necessary. The sections were stained with an aqueous solution of safranin and astra blue, dehydrated, and mounted on glass slides. From each wood cross-section, we usually measured, with an ocular micrometer, the diameters of 25 conduits. For each distance from the tip, we computed the mean conduit diameter (data available in Dataset S1). Some plants, such as arborescent monocots and cycads, have thick stems that lack the readily shed fine twigs of conventional trees. In these species, much of the widening in conduits has been found to be concentrated in the leaves, which are usually large and often have massive, woody petioles and rachises. In these species, the large leaves take the place of sheddable twigs. To be able to include these species, we therefore followed previous practice (70) in tracing tip-to-base conduit widening from the tips of the leaves. The “organ type” column in the dataset indicates when data are from leaves vs. stems.

### Resistance Ratio, Conductance, and Carbon Cost.

Following the definition of total resistance in Eq. **4**, we define the rescaled resistance Ω=R/μK. Upon substituting the profile $\sigma_{PL}\left(h\right)\equiv A\;\sqrt{h/h_{M}}$ for the pure power-law case and Eq. **11** (which we will denote as $\sigma_{WPM}$) into Eq. **4****,** we obtain the following expression for the rescaled resistances:

$${\text{Ω}}_{PL}=\overset{h_{M}}{\underset{h_{0}}{\int }}\frac{1}{\sigma_{PL}^{2}\left(h\right)}dh=\frac{h_{M}}{A^{2}}\ln\frac{h_{M}}{h_{0}}$$ [13]

$${\text{Ω}}_{WPM}=\overset{h_{M}}{\underset{h_{0}}{\int }}\frac{1}{\sigma_{WPM}^{2}\left(h\right)}dh=\frac{h_{M}}{2\sigma_{M}^{2}}\ln\frac{2h_{M}-h_{0}}{h_{0}}.$$ [14]

Recall that $h_{0}$ is the tracheid or vessel element length at the tip of the twig farthest from the base of the plant, and $h_{M}$ is tree height. The ratio of the resistances is

$$\frac{{\text{Ω}}_{PL}}{{\text{Ω}}_{WPM}}=2{\left(\frac{\sigma_{M}}{A}\right)}^{2}\frac{\ln\frac{h_{M}}{h_{0}}}{\ln\frac{2h_{M}-h_{0}}{h_{0}}}.$$ [15]

Our analytical profile clearly leads to lower hydrodynamic resistance than a simple power-law profile with the same boundary conditions ($h_{0},\;h_{M}$) for a given plant. An advantage of our model is that the *W* term does not include carbon costs explicitly. This allows us to compare the relative carbon costs, for a given conductance, of a pure power-law profile versus the WPM profile. While the WPM conductance is markedly higher than a pure power-law profile, the carbon costs of the two profiles are found to be nearly identical (Fig. 3*C*) for the plants studied here. From Eq. **8**, for the power-law profile, we get

$$C_{PL}=\overset{h_{M}}{\underset{h_{0}}{\int }}2\pi \sqrt{\sigma_{PL}\left(h\right)/\pi }dh=\frac{8}{5}h_{M}\sqrt{\pi A}\left(1-{\left(\frac{h_{0}}{h_{M}}\right)}^{5/4}\right),$$ [16]

while, for our optimal prediction, we have the following expression:

$$C_{WPM}=\overset{h_{M}}{\underset{h_{0}}{\int }}2\pi \sqrt{\sigma_{WPM}\left(h\right)/\pi }dh=2h_{M}\sqrt{\pi \sigma_{M}}\overset{1}{\underset{\frac{h_{0}}{h_{M}}}{\int }}{\left(t\left(2-t\right)\right)}^{1/4}dt.$$ [17]

The rescaled whole-tree conductance for our optimal prediction and the power-law profiles is the inverse of the rescaled resistances and is given by

$$Cond_{PL}=\frac{1}{\Omega_{PL}}={\left(\frac{h_{M}}{A^{2}}\ln\frac{h_{M}}{h_{0}}\right)}^{-1}$$ [18]

$$Cond_{WPM}=\frac{1}{\Omega_{WPM}}={\left(\frac{h_{M}}{2\sigma_{M}^{2}}\ln\frac{2h_{M}-h_{0}}{h_{0}}\right)}^{-1}.$$ [19]

Our results show that, for the same carbon cost, the WPM profile has a conductance 1.43 times that of a pure power-law.

### Data Fitting.

We fit our empirical data with the optimal profile Eq. **11** with the nonlinear least-squares solving algorithm *lsqcurvefit* in Matlab (https://it.mathworks.com/help/optim/ug/lsqcurvefit.html). Mathematically, *lsqcurvefit* is equivalent to solving the minimization problem $\underset{z}{\min}\underset{i}{\sum }{\left(f\left(z,x_{i}\right)-y_{i}\right)}^{2}.$ For the *j*th plant, $y_{i}=\sigma_{j}\left(h_{i}^{j}\right)$ is the measured xylem conduit cross-section at distance $x_{i}=h_{i}^{j}$ from the tip, $f\left(x,x_{i}\right)$ is the nonlinear curve Eq. **11**, and $z\equiv \;\sigma_{M}^{j}$ is the free parameter to be fitted. We fit the data for each plant to calculate the value of the unknown parameter$\sigma_{M}^{j}$. We used several initializations of the minimization algorithm to test the robustness of our fits. In addition, we fit the data with another algorithm, the *fminsearch* code of Matlab (https://it.mathworks.com/help/matlab/ref/fminsearch.html), as a further test of the fitting approach. For visual purposes, we made the following transformation of the height axis:

$$X=\;\;\;\sqrt{x},$$ [20]

to better highlight the power-law behavior at small distances from the tip.

## Supplementary Material

Supplementary File

Supplementary File

## Data Availability

The .csv plant trait values data are available in Dataset S1.
