# Supplementary material for: The Widened Pipe Model of plant hydraulic evolution
Source: Proc Natl Acad Sci U S A. 2021 May 26;118(22):e2100314118. doi: 10.1073/pnas.2100314118 (PMC8179198; doi:10.1073/pnas.2100314118)
Supplement: Supplementary File [file pnas.2100314118.sapp.pdf]

PNAS
www.pnas.org

Supplementary Information for

The Widened Pipe Model of plant hydraulic evolution

Loren Koçillari, Mark E. Olson, Samir Suweis, Rodrigo P. Rocha, Alberto Lovison, Franco Cardin, Todd E. Dawson, Alberto Echeverría, Alex Fajardo, Silvia Lechthaler, Cecilia Martínez-Pérez, Carmen Regina Marcati, Kuo-Fang Chung, Julieta A. Rosell, Alí Segovia-Rivas, Cameron B. Williams, Emilio Petrone-Mendoza, Andrea Rinaldo, Tommaso Anfodillo, Jayanth R. Banavar, and Amos Maritan

Corresponding authors: Mark E. Olson, Andrea Rinaldo, and Jayanth R. Banavar
Email: molson@ib.unam.mx, andrea.rinaldo@epfl.ch, or banavar@uoregon.edu

This PDF file includes:

- Supplementary text
- Figure S1
- SI References

Other supplementary materials for this manuscript include the following:

- Dataset S1

Supplementary Information Text

Mathematical derivation of the optimal solution

The minimization of the functional in Eq. (9) of the main text is equivalent to solving the Euler-Lagrange equations corresponding to the Lagrangian in Eq. (10) of the main text, which is identical to the dynamics of a classical particle of mass α, moving in a 1-dimensional potential $V(\sigma) = -1/\sigma^2$ with position $\sigma(h)$ at time h. Thus, the trajectory of the particle is identical to the desired optimal cross-sectional profile of the xylem conduits. This analogy allows one to obtain the solution by using energy conservation, which follows from the form of the functional F itself (1). The energy E of the system is:

$$E = \dot{\sigma}\frac{\partial L}{\partial \dot{\sigma}(h)} - L = \frac{\alpha \dot{\sigma}^2(h)}{2} - \frac{1}{\sigma^2(h)} \quad , \qquad (S1)$$

which is independent of h when $\sigma(h)$ minimizes the functional F in Eq. (9) of the main text. The particle's trajectory is bounded only for $E<0$ and correspondingly $\sigma(h)$ increases monotonically from h_0 till h_M where the motion is reversed. E is the second parameter entering our theory. From Eq. (S1), one gets the following differential equation for the derivative of the xylem profile:

$$\dot{\sigma}(h) = \sqrt{\frac{2}{\alpha}}\sqrt{E + \frac{1}{\sigma^2(h)}} \qquad (S2)$$

The solution of Eq. (S2) is:

$$\sigma(h) = \left(\frac{8}{\alpha}\right)^{1/4}\left[h\left(1 - h\frac{|E|}{\sqrt{2\alpha}}\right)\right]^{1/2} \qquad (S3)$$

where the boundary condition $\sigma(h = 0) = 0$ has been used consistently with the fact that at $h_0 \approx 0$ we have $\sigma(h_0) \approx 0$. Instead of using E and α as free parameters, we introduce two directly measurable quantities such as the plant height:

$$h_M = \sqrt{\frac{\alpha}{2}}\frac{1}{|E|} \qquad (S4)$$

and the corresponding value of the xylem conduit cross-sectional area:

$$\sigma_M = \sigma(h_M) = \frac{1}{\sqrt{|E|}} \qquad (S5)$$

On substituting Eqs. (S4, S5) into Eq. (S3), we get Eq. (11) of the main text.

Optimal Pareto front in objective space

The set of optima is made up of the solutions of multi-objective optimization problems (2–4). In our case, we have two objective functions, the hydraulic resistance R and the widening rate W and thus the optimal front would correspond to a 1-D curve in R-W space spanned by the free parameter α (or equivalently λ). By substituting Eq. (S2) into the widening term (given by Eq. (7) of the main text, with $a_2 = 1$), we get:

$$W_{WPM} = \int_{h_0}^{h_M} \dot{\sigma}^2(h)dh = \frac{2}{\alpha}\left(\Omega_{WPM} + E(h_M - h_0)\right) \quad (S6)$$

where the term Ω_{WPM} is given by Eq. (14) of the main text. Noting that $\alpha = \frac{2h_M^2}{\sigma_M^4}$, and substituting Eq. (S5) into Eq. (S6), we can rewrite Eq. (14) and Eq. (S6) as follows:

$$\Omega_{WPM} = \sqrt{\frac{\alpha}{2}}\, ln \sqrt{\frac{2h_M - h_0}{h_0}} \quad (S7)$$

$$W_{WPM} = \sqrt{\frac{2}{\alpha}}\left(ln \sqrt{\frac{2h_M - h_0}{h_0}} - 1 + \frac{h_0}{h_M}\right) \quad (S8)$$

We get the following analytical expression of the Pareto front in the $ln\,\Omega - ln\,W$ objective space:

$$ln\Omega_{WPM} = -lnW_{WPM} + J \quad (S9)$$

where J is defined as:

$$J = +\ln\left(ln \sqrt{\frac{2h_M - h_0}{h_0}} - 1 + \frac{h_0}{h_M}\right) + ln\, ln \sqrt{\frac{2h_M - h_0}{h_0}} \quad (S10)$$

The costs Ω and W should be analyzed as the free parameter $\alpha = 2h_M^2/\sigma_M^4$ (or equivalently λ) varies for plants with the same h_M (h_0 is relatively constant within individuals and is treated as fixed). From Eqs. (S7-S8) we get $\Omega W =$ constant, corresponding to a hyperbola in the $\Omega - W$ plane. However, note that there is a weak logarithmic dependence on h_M in Eq. (S7) and (S8), which is weaker than the α dependence. This is also evident in the form given by Eq. (S10), where the weak dependence on h_M is displayed. Figure 4 of the main text shows the optimal front in the $ln\Omega - lnW$ plane for the 102 plants measured. Were J in Eq. (S10) constant, the optimal front ought to be a line of slope -1 in the lnΩ - ln$W$ plane. However due to the extremely

weak dependence of J on h_M, the line of slope -1 becomes a narrow strip of non-zero width.

SI Figures

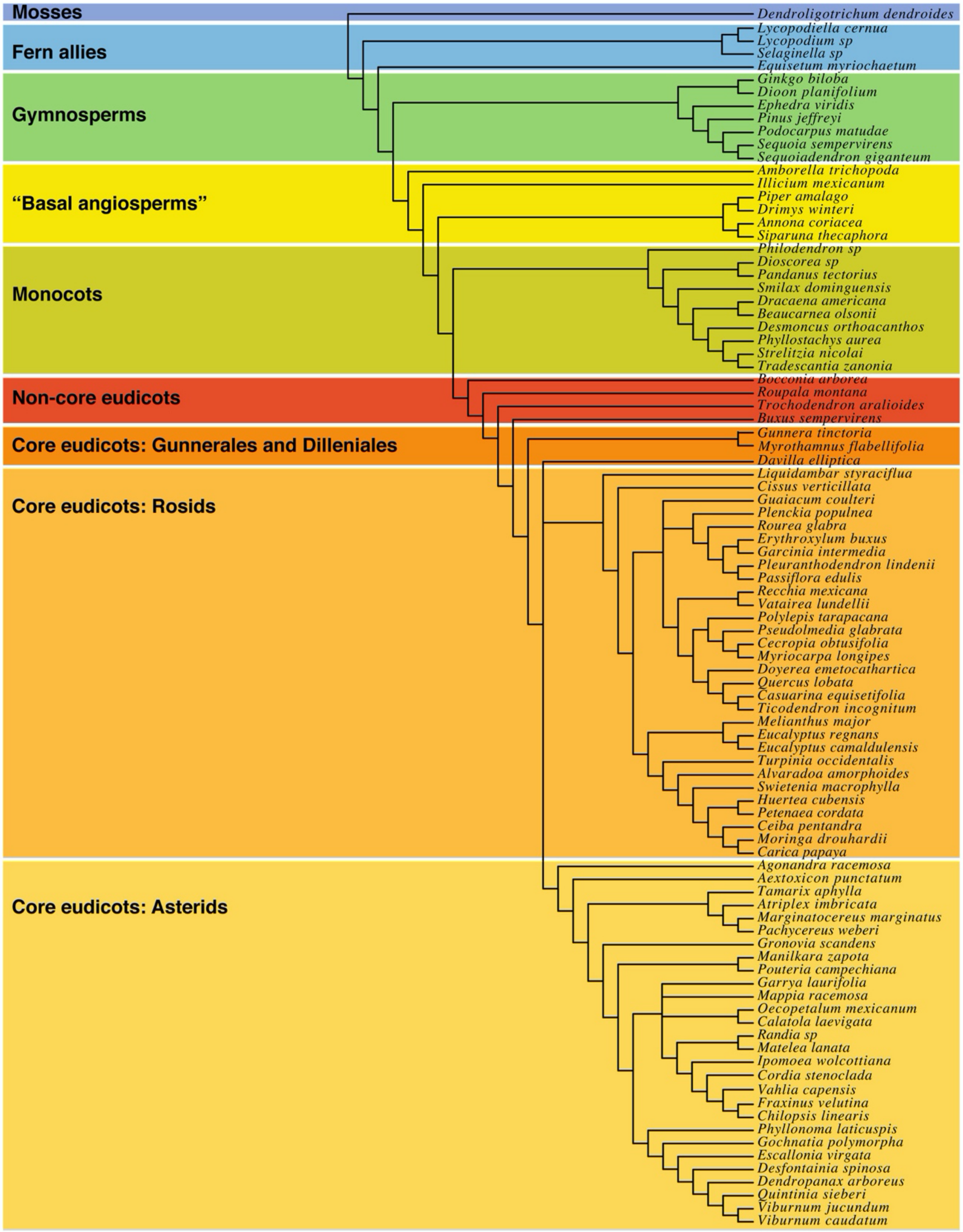

Figure S1. Phylogenetic coverage of the sampled species. Our phylogenetic sampling covers virtually all orders of terrestrial vascular plants. Tree based on published backbone phylogenies (5, 6).

Dataset S1 (separate file)
Species, authorities, distances from the stem tip from which each sample was collected, mean conduit diameter at each distance from the stem tip, organ type sampled (whether the conduits were measured in leaf, stem, or root), and vessels per group in longitudinal profiles of tip-to-base conduit widening.

SI References

1. H. Goldstein, C. Poole, J. Safko, Classical mechanics (2002).

2. J. Branke, J. Branke, K. Deb, K. Miettinen, R. Słowiński, *Multiobjective optimization: Interactive and evolutionary approaches* (Springer Science & Business Media, 2008).

3. V. Pareto, *Cours d'économie politique* (Librairie Droz, 1964).

4. O. Shoval, *et al.*, Evolutionary trade-offs, Pareto optimality, and the geometry of phenotype space. *Science* **336**, 1157–1160 (2012).

5. D. E. Soltis, *et al.*, Angiosperm phylogeny: 17 genes, 640 taxa. *American journal of botany* **98**, 704–730 (2011).

6. M. W. Chase, *et al.*, An update of the Angiosperm Phylogeny Group classification for the orders and families of flowering plants: APG IV. *Botanical Journal of the Linnean Society* **181**, 1–20 (2016).
